# Unraveling the Relation of Parkinson's Disease and Metabolites: A Combined Analysis of Stool and Plasma Metabolites Based on Untargeted Metabolomics Technology

**DOI:** 10.1111/cns.70424

**Published:** 2025-05-16

**Authors:** Sufang Liu, Qiang Zhao, Jie Tang, Xianhong Li, Juan Wang, Yuting Zhao, Zhengting Yang, Xin Pan, Rui Xiang, Jing Tian, Puqing Wang

**Affiliations:** ^1^ Department of Neurology Xiangyang No. 1 People's Hospital, Hubei University of Medicine Xiangyang Hubei China; ^2^ Hubei Provincial Clinical Research Center for Parkinson's Disease, Xiangyang Key Laboratory of Movement Disorders Xiangyang No. 1 People's Hospital, Hubei University of Medicine Xiangyang Hubei China

**Keywords:** biomarkers, intestinal inflammation, intestinal permeability, Parkinson's disease, untargeted metabolomics

## Abstract

**Objective:**

Metabolomics technology has been widely utilized to uncover the action mechanisms of Parkinson's Disease (PD) and to identify PD‐related biomarkers. In this study, we compared plasma and fecal metabolite levels between PD patients and their healthy spouses (HS), aiming to identify the associations of differential metabolites with intestinal inflammation, intestinal barrier function, and clinical characteristics of PD.

**Methods:**

Untargeted metabolomics techniques were used to characterize plasma and fecal metabolite profiles. We identified metabolites with elevated plasma levels in PD patients, while no significant differences were observed in fecal samples. Partial correlation analysis was employed to investigate the associations between these metabolites, markers of intestinal inflammation (calprotectin and lactoferrin), markers of intestinal permeability (α‐1‐antitrypsin and zonulin), and clinical characteristics of PD patients.

**Results:**

The study identified ten metabolites that were significantly elevated in the plasma of PD patients compared to HS (*p* < 0.05), while their fecal concentrations did not differ significantly. Correlation analysis revealed that elevated levels of differential metabolites in the plasma of PD patients were associated with increased intestinal permeability and inflammation. Furthermore, five metabolites, including 3,4‐Dihydroxyphenylglycol O‐sulfate and Propyl gallate, were linked to PD symptoms. Receiver Operating Characteristic (ROC) curves demonstrated that these metabolites could effectively distinguish between PD patients and HS, with an area under the curve (AUC) of 0.94, indicating excellent predictive performance.

**Conclusions:**

This study identified significant metabolite alterations in PD patients and revealed their associations with intestinal barrier dysfunction and clinical characteristics of the disease.

## Graphical abstract

By performing untargeted metabolomics analysis on plasma and fecal samples from Parkinson's disease patients and their healthy spouses, potential biomarkers with good predictive value were identified, and their possible underlying mechanisms were explored.

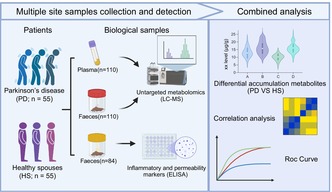

## 1 Introduction

Parkinson's Disease (PD) is a rapidly growing neurodegenerative disorder worldwide in terms of both mortality and prevalence. According to the Global Burden of Disease study, approximately 6.1 million people suffer from PD globally [1]. PD is clinically characterized by motor symptoms such as static tremor, slow movement, rigidity, and postural instability [2]. Notably, many PD patients experience non‐motor symptoms, such as sleep disorders, reduced sense of smell, and persistent constipation, up to 10–20 years before the onset of motor symptoms, with constipation being particularly prevalent [3]. Prospective studies have reported that individuals with chronic constipation have a 6–to 19‐fold increased risk of developing PD within 10 years [4, 5]. In 2003, Braak [6] first proposed the gut‐brain axis hypothesis, which was later corroborated by Sangjune Kim et al. [7] in 2019 through animal studies, highlighting the role of a healthy intestinal environment in maintaining metabolite homeostasis and intestinal barrier integrity. Our previous clinical research identified that there were significant differences in intestinal microbes between PD patients and healthy spouses (HS), and these differences were correlated with clinical characteristics [8, 9, 10]. However, research reports on the mechanisms behind the roles of various microorganisms in PD often show inconsistencies. Some scholars have proposed that metabolites, as important intermediates between microorganisms and hosts, can play a role in the pathogenesis of PD by promoting the pro‐inflammatory environment, oxidative stress, and calcium homeostasis disruption in the gut [11, 12]. For example, Uric acid is involved in the oxidative stress pathway [13, 14], tryptophan is related to energy metabolism [15], lipopolysaccharide can enhance pro‐inflammatory response [16], and short‐chain fatty acids regulate intestinal permeability [17, 18, 19]. However, conclusions are disputed in different studies.

Metabolomic analysis using liquid chromatography‐mass spectrometry (LC–MS) has recently gained recognition for its role in understanding the human gut microbiome [20]. This approach can reflect the pathophysiological process of the diseases by revealing the overall trajectory of metabolite changes in the body under the influence of intrinsic and extrinsic factors. Studies on the gut microbiome‐metabolome relationship in PD patients have typically focused on single‐sample analyses of either stool or plasma [21]. Recent studies have shown that while short‐chain fatty acids (SCFAs) are present in low concentrations in stool, they are elevated in plasma, possibly due to compromised intestinal barrier function [18, 19]. Epidemiological studies indicate that individuals with inflammatory bowel disease face a significantly higher risk of PD compared to healthy individuals [22]. A stable intestinal environment is crucial for maintaining barrier integrity, and its disruption can cause an “intestinal leakage phenomenon” and aggravate the systemic inflammatory response [23]. David Devos [24] conducted biopsies on the intestinal tissues of participants in the trial and revealed significantly elevated intestinal inflammation in PD patients. Additionally, studies by Forsyth and Salat‐Foix [25, 26] using oral administration of unmetabolized sugars showed that PD patients demonstrated increased intestinal permeability. Elevated levels of established markers of intestinal inflammation and permeability, such as α‐1‐antitrypsin, zonulin, calprotectin, and lactoferrin, were found in the stools of PD patients [27, 28]. These findings underscore the potential role of intestinal metabolites, inflammation, and permeability in PD's pathophysiology.

Based on the above information, we hypothesize that if metabolite levels in the gut remain relatively stable but are elevated in plasma, this may be due to their translocation into the bloodstream through a compromised intestinal barrier. To investigate this, we collected plasma and fecal samples from PD patients and HS simultaneously. Using the untargeted metabolomics approach of LC–MS, we studied the differences in plasma and fecal metabolite levels between the two groups, and explored the relationship between the differential metabolites and markers of intestinal inflammation and permeability with a goal of identifying reliable biomarkers and elucidating their potential mechanisms in the development of PD.

## 2 Methods

### 2.1 Participant Inclusion

We recruited 55 PD patients from the Department of Neurology of Xiangyang First People's Hospital, affiliated with Hubei University of Medicine, as the clinical trial group. The diagnoses of PD were confirmed by two specialists in Parkinson's disease according to the Movement Disorder Society Clinical Diagnostic Criteria for PD [29]. Simultaneously, we recruited 55 HS who have been living with the PD patients for a long time to serve as the healthy control group. This approach was to minimize potential confounding factors such as diet, lifestyle, and living conditions.

Patient exclusion criteria: (1) Parkinsonism‐plus syndrome or secondary Parkinsonism syndrome; (2) inflammatory bowel syndrome; (3) psychiatric illness; (4) diabetes, gastrointestinal disease, surgical history, or infectious diseases; (5) antibiotics/probiotics used for nearly 3 months.

Healthy spouses (HS, *n* = 55) lived in the same household with PD patients. Exclusion criteria for healthy spouses were as follows: (1) obvious digestive diseases (history of gastrointestinal surgery or severe infection); (2) psychiatric illness and neurodegenerative disease; (3) use of antibiotics/probiotics for nearly 3 months; and (4) history of going out of Xiangyang city (> 5 days) in the last 6 months.

### 2.2 Clinical Characteristics Evaluation

In addition to demographic data, such as height, weight, gender, Body Mass Index (BMI), and Rapid Eating Assessment for Participants—Shortened Version (REAP‐S), we assessed various clinical characteristics, including olfactory function, sleep quality, constipation, cognitive function, and quality of life. These evaluations were conducted using the Hamilton Anxiety Scale (HAMA), Hamilton Depression Scale (HAMD), Montreal Cognitive Assessment (MOCA), Hyposmia rating scale, Rome III Criteria for Functional Constipation (ROMA‐III), REM Sleep Behavior Disorder Screening Questionnaire (RBDSQ), Non‐Motor Symptoms Questionnaire (NMSQ), and the 39‐item Parkinson's Disease Questionnaire (PDQ‐39). Thirty‐seven PD patients underwent Motor Symptoms Evaluation using the Movement Disorder Society Unified Parkinson's Disease Rating Scale (MDS‐UPDRS) Part III and Hoehn & Yahr (H & Y) staging during the “off” period to assess the severity of motor symptoms. The daily dosage of PD medication was converted into the levodopa equivalent daily dose (LEDD) [30]. All participants provided written informed consent, and the study was approved by the Ethics Committee of Xiangyang First People's Hospital.

### 2.3 Collection and Processing of Biological Samples

(1) Blood samples were collected in the morning after participants fasted for at least 10 h. Fasting venous blood was obtained via direct venipuncture into purple vacuum tubes containing EDTA anticoagulant. The samples were then centrifuged at 4°C for 10 min at 3000 rpm to ensure the plasma was free of hemolysis. The upper layer of plasma was carefully extracted using a sterile technique, aliquoted into sterile tubes, and stored at −80°C until further analysis. (2) Fecal samples were collected in sterile containers and subsequently transferred into sterile tubes using a sterile scoop, snap‐frozen with liquid nitrogen, and then stored in a −80°C refrigerator. To prevent changes in metabolite levels, the collection and treatment of feces were controlled within 1 h. In this study, plasma and fecal samples from 55 PD patients and 55 HS were collected for untargeted metabolomics analysis. However, due to sample contamination and insufficient quantity, intestinal permeability and inflammatory markers were only tested in 42 stool samples from both the PD and HS groups.

### 2.4 Metabolomics Detection

Waters UPLC I‐Class Plus (Waters, USA) in tandem with Q Exactive high‐resolution mass spectrometer (Thermo Fisher Scientific, USA) was used for the separation and detection of metabolites. Metabolite extracts were analyzed using a BEH C18 column under gradient elution conditions. For positive ion mode, the mobile phases included 0.1% formic acid in aqueous solution and 0.1% formic acid in methanol. For negative ion mode, the mobile phases were 10 mM ammonium formate in aqueous solution and 10 mM ammonium formate in 95% methanol. Both primary and secondary mass spectrometry data acquisition were performed using the Q Exactive mass spectrometer. Detailed chromatographic and mass spectrometry conditions are provided in the Supporting Information.

Data processing was performed using Compound Discoverer 3.3 software (Thermo Fisher Scientific, USA), in conjunction with the BGI Metabolome Database (BMDB). Following data analysis with BMDB, mzCloud, and ChemSpider databases, a data matrix containing metabolite peak areas and identification results was generated. This matrix was subsequently imported into metaX for data preprocessing, with normalization carried out using the Probabilistic Quotient Normalization (PQN) method. The relative peak areas were calculated, and batch effects were corrected using Quality Control‐based Robust LOESS Signal Correction (QC‐RLSC). Compounds with a coefficient of variation (CV) greater than 30% in relative peak area across all QC samples were excluded from the analysis.

### 2.5 Inflammatory and Permeability Markers Measurements

The markers of intestinal inflammation (calprotectin/lactoferrin) concentrations were determined by enzyme‐linked immunosorbent assay (ELISA) kit (F9913‐A/F1241‐A, Fankew, Shanghai Kexing Trading Co. Ltd). Markers of intestinal permeability in fecal samples were measured using the Zonulin ELISA Research Kit and Alpha‐1‐antitrypsin ELISA Research Kit (F10869‐A and F10535‐A, Fankew, Shanghai Kexing Trading Co. Ltd) following the respective protocols provided by the manufacturer.

### 2.6 Combined Analysis

A combined analysis of the plasma and fecal metabolites was performed on two participant groups (*n* = 110). Metabolites that were significantly increased in the plasma of PD patients but not elevated in the feces compared to HS were identified. These differential metabolites were then integrated with the biomarker protein levels (*n* = 84) and clinical parameters. Metabolites that showed a significant positive correlation with both the biomarker proteins and clinical features were selected. These metabolites were subsequently used as features to develop a predictive model for PD.

### 2.7 Statistical Analysis

The Shapiro–Wilk test was used to assess the normality of the data, while the Levene test was used to evaluate the homogeneity of variance. For continuous variables, the mean ± standard deviation was expressed. Two samples with normal distribution and homogeneity of variance were compared using the *T*‐test. For non‐normally distributed data, the Wilcoxon rank sum test was applied. To control the false discovery rate (FDR) caused by multiple testing, the Benjamini‐Hochberg method was used, with FDR < 0.05 considered statistically significant. Categorical variables were presented as frequencies (percentages), and the differences between the two groups were compared using the chi‐square test (*p* < 0.05 was considered significant). The correlation between the differential metabolites and marker proteins was evaluated by the nonparametric Spearman rank correlation coefficient. Partial correlation analysis between differential metabolites and clinical parameters was performed, and corrections were made for age, BMI, and LEDD (FDR < 0.05 was considered significant). Correlation analysis and heat maps were generated using the corrplot package in R (version 0.92). The differential metabolites that were significantly positively correlated with both marker proteins and clinical features were used as features to construct a Parkinson's disease prediction model. The model was developed using the random forest algorithm in sklearn (n_estimators = 150, random_state = 1), and the model was validated using five‐fold cross‐validation.

Demographic analysis was performed using IBM SPSS Statistics version 25.0.0 (SPSS Inc., Chicago, IL, USA), and the significance levels were set at 0.05 (2‐tailed). All statistical analyses in this study, except for demographic analysis, were performed in the Anaconda 202,303 Python package pingouin (version 0.5.3), and all graphics were generated by R (version 4.1.3).

## 3 Results

### 3.1 The Demographics and Clinical Characteristics of Participants

Due to the incomplete basic information of 5 patients, we only collected and analyzed the basic information of 50 PD patients and 50 HS. The basic demographic characteristics, including age and BMI, were similar between PD patients and HS. The dietary assessment scale (REAP‐S) showed no differences in diet between the two groups. In the PD group, the mean H&Y stage was 2.61 ± 0.91, with a disease duration of 6.45 ± 4.02 years; the MDS‐UPDRS part III score was 46.83 ± 17.06. Additional clinical characteristics of study participants are summarized in Table 1.

**TABLE 1:** Demographic and clinical characteristics of participants.

	Patients with PD (*N* = 50)	Healthy spouses (HS) (*N* = 50)	*p*
Age, years	67.30 ± 6.13	66.78 ± 7.50	0.705
Sex, male (*n*, %)	31 (62.0)	30 (60.0)	0.838
Height (cm)	1.65 ± 0.06	1.626 ± 0.06	0.083
Weight (Kg)	63.43 ± 8.62	62.46 ± 8.56	0.574
BMI (kg/m^2^)	23.40 ± 3.05	23.64 ± 3.20	0.701
REAP‐S (mean)	32.56 ± 2.71	32.26 ± 2.47	0.565
Disease duration (y)	6.45 ± 4.02	NA	
LEDD (mg)	583.45 ± 402.44	NA	
MDS‐UPDRS Part I score	12.68 ± 6.61	NA	
MDS‐UPDRS Part II score	16.98 ± 9.58	NA	
MDS‐UPDRS Part III score	46.83 ± 17.06	NA	
HAMA score	7.88 ± 7.27	NA	
HAMD score	9.00 ± 6.64	NA	
MoCA score	19.92 ± 6.13	NA	
ROMA‐III, Y(*n*, %)	32 (64.0)	NA	
Hyposmia rating scale	17.35 ± 8.36	NA	
H&Y stage	2.61 ± 0.91	NA	
RBDSQ	4.34 ± 3.33	NA	
NMSQ score	12.68 ± 5.24	NA	
PDQ‐39	41.40 ± 22.85	NA	

*Note:* Data are shown as mean ± standard deviation or *n* (%). Comparisons between groups were assessed with the Student's *t*‐test or Mann–Whitney *U* test for quantitative variables and the Chi‐Squared test for categorical variables. *p* value represents where differences in the characteristics between PD and HS were detected.

Abbreviations: BMI, body mass index; HAMA, Hamilton Anxiety Scale; HAMD, Hamilton Depression Scale; H&Y stage, Hoehn and Yahr stage; LEDD, levodopa equivalent daily dose; MDS‐UPDRS, Movement Disorder Society‐sponsored revision of the Unified Parkinson's Disease Rating Scale; MoCA, Montreal cognitive assessment; NA, not available; NMSQ, Non‐Motor Symptoms Questionnaire; PD, Parkinson's disease; PDQ‐39, The 39‐item Parkinson's Disease Questionnaire; RBDSQ, REM Sleep Behavior Disorder Screening Questionnaire; REAP‐S, Rapid Eating Assessment for Participants—Shortened Version; ROMA‐III, Rome III Criteria for Functional Constipation.

### 3.2 Comparison of Plasma and Fecal Metabolite Levels Between PD and HS

**FIGURE 1:**
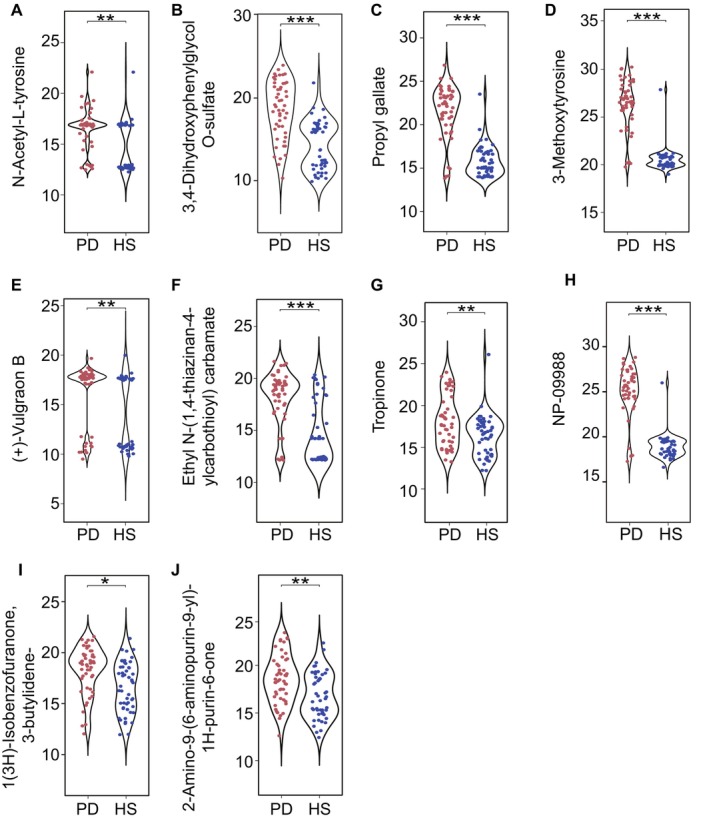
Differential abundance of plasma metabolites between the PD and HS groups is visualized through violin plots. The plots illustrate the distribution patterns of ten significantly altered metabolites: (A) N‐Acetyl‐L‐tyrosine, (B) 3,4‐Dihydroxyphenylglycol O‐sulfate, (C) Propyl gallate, (D) 3‐Methoxytyrosine, (E) (+)‐Vulgraon B, (F) Ethyl N‐(1,4‐thiazinan‐4‐ylcarbothioyl)carbamate, (G) Tropinone, (H) NP‐019988, (I) 1(3H)‐Isobenzofuranone,3‐butylidene‐, and (J) 2‐Amino‐9‐(6‐aminopurin‐9‐yl)‐1H‐purin‐6‐one. Statistical significance is denoted as follows: *FDR < 0.05, **FDR < 0.01, ***FDR ≤ 0.001.

We performed untargeted metabolomics analysis on 110 plasma samples (PD = 55, HS = 55) and 110 fecal samples (PD = 55, HS = 55) from all participants, and compared their metabolites between PD patients and HS using *t*‐tests. Forty‐five plasma metabolites were identified to be significantly elevated in PD patients compared to HS (*p* < 0.05). Among these, 10 metabolites, including 3‐Methoxytyrosine, Tropinone, N‐Acetyl‐L‐tyrosine, Propyl gallate, and (+)‐Vulgraon B, were significantly overexpressed in the plasma compared with the HS group, but there was no significant difference in the fecal samples between the two groups (Table 2, Figure 1).

**TABLE 2:** Metabolites were significantly elevated in plasma but not in feces between PD and HS.

Metabolite	*p*	*FDR* ^*a*^
3‐Methoxytyrosine	1.16e‐20	7.05e‐20
Tropinone	9.32e‐05	0.00
N‐Acetyl‐L‐tyrosine	1.89e‐05	0.00
Propyl gallate	2.17e‐18	1.99e‐16
3,4‐Dihydroxyphenylglycol O‐sulfate	4.97e‐19	6.08e‐17
1 (3H)‐Isobenzofuranone, 3‐butylidene—	0.00	0.02
2‐Amino‐9‐(6‐aminopurin‐9‐yl)‐1H‐purin‐6‐one	0.00	0.00
NP‐019988	1.55e‐21	4.69e‐19
Ethyl N‐(1,4‐thiazinan‐4‐ylcarbothioyl) carbamate	6.10e‐09	6.16e‐07
(+)‐Vulgraon B	8.81e‐05	0.00

^a^ FDR is controlled by the Benjamini–Hochberg method.

### 3.3 Comparison of Intestinal Permeability and Intestinal Inflammation Markers Between PD and HS

**FIGURE 2:**
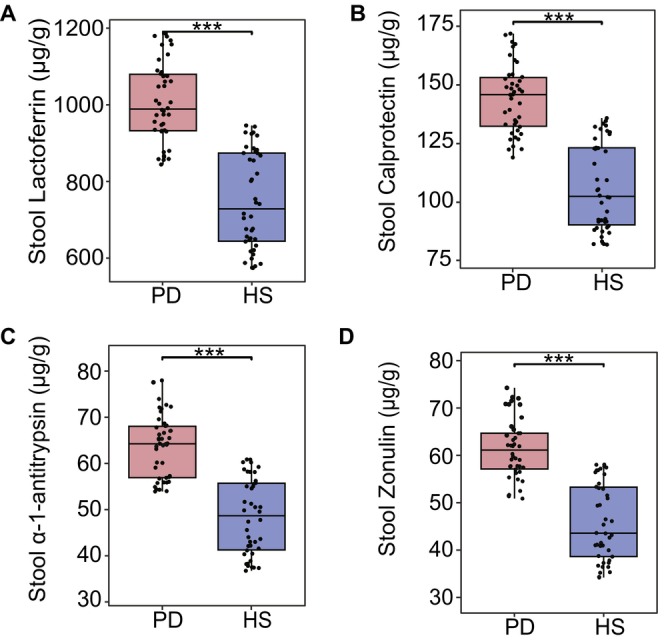
Comparative analysis of intestinal inflammation and permeability biomarkers in PD patients and HS subjects. (A, B) Quantification of intestinal inflammation biomarkers: (A) lactoferrin (FDR = 1.64 × 10^−11^) and (B) calprotectin (FDR = 1.64 × 10^−11^). (C, D) Assessment of intestinal barrier integrity markers: (C) α‐1‐antitrypsin (FDR = 1.33 × 10^−10^) and (D) zonulin (FDR = 2.10 × 10^−11^). Statistical significance is denoted as ***FDR ≤ 0.001.

The ten metabolites that were significantly elevated in the plasma of PD patients but not in their feces may reflect increased intestinal permeability due to compromised intestinal barrier damage in these patients. To further assess intestinal barrier integrity, we measured markers of intestinal permeability and inflammation. Some fecal samples were insufficient in quantity due to contamination and other factors. Therefore, we only used 84 samples (PD = 42, HS = 42) from the above‐mentioned participants for the measurement of biomarkers. Significant elevations were observed in intestinal inflammatory markers in the PD group compared to HS. Specifically, lactoferrin levels (PD: 1003.383 μg/g vs. HS: 748.077 μg/g, FDR < 0.01; Figure 2A) and calprotectin (PD: 143.863 μg/g vs. HS: 106.368 μg/g, FDR < 0.01; Figure 2B) were significantly elevated in the PD group. Similarly, levels of intestinal permeability markers α‐1‐antitrypsin (PD: 63.689 μg/g vs. HS: 48.444 μg/g, FDR < 0.01; Figure 2C) and zonulin (PD: 61.320 μg/g vs. HS: 45.811 μg/g, FDR < 0.01; Figure 2D) were significantly higher in PD patients compared to HS.

**FIGURE 3:**
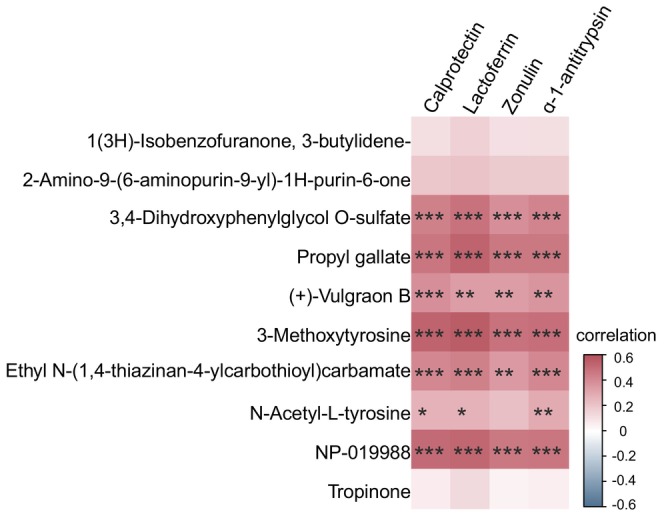
Spearman correlation analysis between differentially abundant plasma metabolites and intestinal biomarkers visualized as a heatmap. The correlation matrix depicts the strength and direction of associations, with red indicating positive coefficients and blue indicating negative coefficients. The statistical significance of correlations is denoted as follows: *p < 0.05, **p < 0.01, ***p < 0.001.

Spearman correlation analysis was conducted to examine the relationship between the ten differential plasma metabolites and markers of intestinal permeability and inflammation in the feces of PD patients. The results indicated that levels of seven plasma metabolites in PD patients—including 3,4‐Dihydroxyphenylglycol O‐sulfate, Propyl gallate, (+)‐Vulgraon B, 3‐Methoxytyrosine, Ethyl N‐(1,4‐thiazinan‐4‐ylcarbothioyl) carbamate, NP‐019988, and N‐Acetyl‐L‐tyrosine—were significantly correlated with increased intestinal permeability and inflammation(Table 3, Figure 3).

**TABLE 3:** Association of differential metabolites with markers of intestinal permeability and inflammation levels.

Metabolite	Protein^a^	R^b^	*p*
3,4‐Dihydroxyphenylglycol O‐sulfate	Calprotectin	0.43	0.00
	Lactoferrin	0.49	0.00
	Zonulin	0.40	0.00
	ɑ‐1‐antitrypsin	0.42	0.00
Propyl gallate	Calprotectin	0.48	0.00
	Lactoferrin	0.53	0.00
	Zonulin	0.47	0.00
	ɑ‐1‐antitrypsin	0.46	0.00
(+)‐Vulgraon B	Calprotectin	0.39	0.00
	Lactoferrin	0.34	0.00
	Zonulin	0.34	0.00
	ɑ‐1‐antitrypsin	0.36	0.00
3‐Methoxytyrosine	Calprotectin	0.53	0.00
	Lactoferrin	0.56	0.00
	Zonulin	0.48	0.00
	ɑ‐1‐antitrypsin	0.50	0.00
Ethyl N‐(1,4‐thiazinan‐4‐ylcarbothioyl) carbamate	Calprotectin	0.41	0.00
	Lactoferrin	0.44	0.00
	Zonulin	0.36	0.00
	ɑ‐1‐antitrypsin	0.41	0.00
N‐Acetyl‐L‐tyrosine	Calprotectin	0.27	0.02
	Lactoferrin	0.27	0.02
	ɑ‐1‐antitrypsin	0.30	0.01
NP‐019988	Calprotectin	0.51	0.00
	Lactoferrin	0.52	0.00
	Zonulin	0.46	0.00
	ɑ‐1‐antitrypsin	0.48	0.00

^a^ Protein, Markers of intestinal permeability and inflammation.

^b^ *R*, The Spearman's rank coefficient of correlation.

### 3.4 Correlations Between Differential Plasma Metabolites and Clinical Characteristics in PD Patients

**FIGURE 4:**
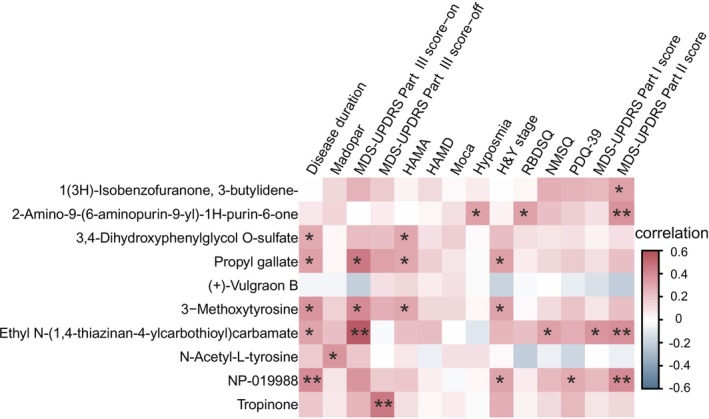
Spearman correlation analysis between differentially abundant plasma metabolites and clinical characteristics of Parkinson's disease, represented as a heatmap. The correlation matrix illustrates the strength and directionality of associations, where red denotes positive correlation coefficients and blue indicates negative correlation coefficients. Statistical significance levels are denoted as follows: *p < 0.05, **p < 0.01, ***p < 0.001.

Given that 3‐Methoxytyrosine levels might be influenced by anti‐Parkinson's medications, we focused on the remaining six differential metabolites and their associations with clinical features. A partial correlation analysis was conducted, adjusting for age, BMI, and LEDD (Figure 4). The associations between differential metabolites and whether PD patients experienced constipation are summarized in Table 4. We found that 3,4‐Dihydroxyphenylglycol O‐sulfate, Propyl gallate, NP‐019988, and Ethyl N‐(1,4‐thiazinan‐4‐ylcarbothioyl) carbamate were significantly positively correlated with disease duration. Additionally, Propyl gallate showed positive correlations with constipation, HAMA, MDS‐UPDRS part III (on), and H&Y stage. Ethyl N‐(1,4‐thiazinan‐4‐ylcarbothioyl) carbamate was positively correlated with MDS‐UPDRS part I, II, and III(on) scores, as well as the NMSQ. The concentration of NP‐019988 correlated with the constipation, H&Y stage, PDQ‐39, and MDS‐UPDRS part II. Detailed correlation results are provided in the Supporting Information (Table 5). (+)‐Vulgraon B showed no significant correlation with any of the measures and therefore will not be included in subsequent studies.

**TABLE 4:** Analysis of the significance of differential metabolites between patients with constipation and those without constipation.

Metabolite	*p*	FDR^a^
3,4‐Dihydroxyphenylglycol O‐sulfate	0.02	0.03^a^
Propyl gallate	0.00	0.01^a^
NP‐019988	0.00	0.01^a^
Ethyl N‐(1,4‐thiazinan‐4‐ylcarbothioyl)carbamate	0.04	0.06
N‐Acetyl‐L‐tyrosine	0.09	0.11
(+)‐Vulgraon B	0.38	0.38

*Note:* Relationships between differential metabolites with constipated or not in PD compared by Wilcoxon rank sum test.

^a^ *FDR* ≤ 0.05; Constipation (*n* = 27), Non‐constipation (*n* = 15).

**TABLE 5:** Correlations between differential metabolites and clinical characteristics in PD patients.

Metabolite	Feature	*R* ^a^	*p*
3,4‐Dihydroxyphenylglycol O‐sulfate	Disease duration	0.30	0.04
Propyl gallate	Disease duration	0.33	0.02
Ethyl N‐(1,4‐thiazinan‐4‐ylcarbothioyl)carbamate	Disease duration	0.32	0.03
NP‐019988	Disease duration	0.41	0.00
N‐Acetyl‐L‐tyrosine	Madopar	0.37	0.01
Propyl gallate	MDS‐UPDRS part III	0.46	0.02
Ethyl N‐(1,4‐thiazinan‐4‐ylcarbothioyl)carbamate	MDS‐UPDRS part III	0.57	0.00
3,4‐Dihydroxyphenylglycol O‐sulfate	HAMA	0.30	0.04
Propyl gallate	HAMA	0.31	0.03
Propyl gallate	H&Y stage	0.33	0.02
NP‐019988	H&Y stage	0.32	0.03
Ethyl N‐(1,4‐thiazinan‐4‐ylcarbothioyl)carbamate	NMSQ	0.33	0.03
NP‐019988	PDQ‐39	0.30	0.04
Ethyl N‐(1,4‐thiazinan‐4‐ylcarbothioyl)carbamate	MDS‐UPDRS part I	0.37	0.01
Ethyl N‐(1,4‐thiazinan‐4‐ylcarbothioyl)carbamate	MDS‐UPDRS part II	0.39	0.01
NP‐019988	MDS‐UPDRS part II	0.41	0.00

Abbreviations: H&Y stage, Hoehn and Yahr stage; HAMA, Hamilton Anxiety Scale; MDS‐UPDRS, Movement Disorder Society‐sponsored revision of the Unified Parkinson's Disease Rating Scale; NMSQ, Non‐Motor Symptoms Questionnaire; PDQ‐39, The 39‐item Parkinson's Disease Questionnaire.

^a^ *R*: The Partial coefficient of correlation.

### 3.5 Discriminant Model Establishment

**FIGURE 5:**
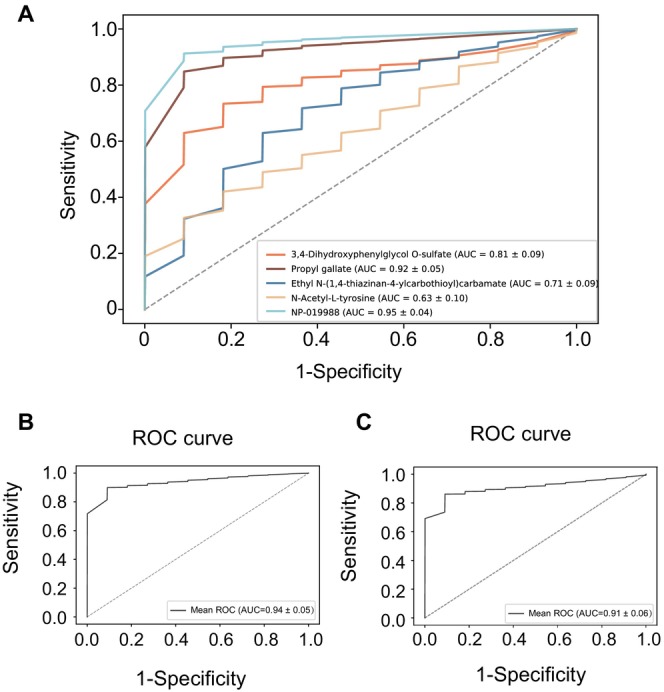
Diagnostic performance evaluation of plasma metabolite biomarkers using Receiver Operating Characteristic (ROC) curves for Parkinson's disease prediction. (A) Comparative ROC curve analysis of individual metabolites as standalone biomarkers. (B) ROC curve analysis of a multi‐metabolite prediction model incorporating 5 metabolites (NP‐019988, Ethyl N‐(1,4‐thiazinan‐4‐ylcarbothioyl)carbamate, 3,4‐Dihydroxyphenylglycol O‐sulfate, Propyl gallate, and N‐Acetyl‐L‐tyrosine), achieving an Area Under the Curve (AUC) of 0.94. (C) ROC curve analysis of a simplified two‐metabolite model (Propyl gallate and N‐Acetyl‐L‐tyrosine), demonstrating an AUC of 0.91.

To further explore the potential clinical utility of the identified metabolites, we constructed a predictive model for PD using the five remaining differential metabolites. The model achieved an area under the curve (AUC) of 0.94, demonstrating excellent predictive performance (Figure 5A). Although two of these metabolites, Propyl gallate and N‐Acetyl‐L‐tyrosine, have been reported in other diseases, they still exhibited strong discriminatory power for distinguishing PD patients from healthy controls, with an AUC of 0.91 (Figure 5B).

## 4 Discussion

This study is the first to employ untargeted metabolomics to identify metabolites that showed no differences in stool samples between PD patients and HS, but were significantly elevated in the plasma of PD patients and investigated the relationships between intestinal permeability, inflammation levels, clinical characteristics, and these differential metabolites. We identified ten metabolites that were exclusively elevated in the plasma of PD patients. Among these, seven metabolites were linked to increased intestinal permeability and higher inflammation levels. Notably, markers of intestinal permeability and inflammation were significantly higher in PD patients compared to HS. This supports our hypothesis that the differences in the metabolites mentioned above may be due to entry into the bloodstream through the damaged intestinal barrier. Furthermore, we developed a predictive model using five metabolites, including Propyl gallate and N‐cetyl‐L‐tyrosine, achieving a diagnostic accuracy of 94% for PD. This model shows significant potential for clinical application in the future.

The intestinal barrier is a regulatory barrier formed by tight junctions between intestinal epithelial cells. Located between the bloodstream and the contents of the intestine, this barrier serves several essential functions: it prevents harmful substances from entering the bloodstream, supports the absorption of nutrients, and aids in eliminating waste [31]. In a longitudinal cohort study, Villumsen et al. [32] revealed that patients with inflammatory bowel disease were at higher risk of developing PD after long‐term follow‐up. Based on this, it is proposed that PD may be initiated by damage to the intestinal barrier, triggering inflammation‐induced increases in intestinal permeability that could lead to greater exposure of the enteric nervous system (ENS) to the microbiome and its toxic products [33].

PD is also known for Inflammatory crosstalk between the gut‐brain axis pathway [34, 35]. Fecal calprotectin and lactoferrin serve as sensitive markers of intestinal inflammation, as they are resistant to enzymatic degradation [36]. The level of α‐1‐antitrypsin in feces indicates protein loss within the intestinal lumen and serves as an indirect measure of mucosal barrier integrity. Zonulin, which regulates intestinal permeability by modulating tight junction function, is another important marker of intestinal permeability in the feces [19]. We observed significantly higher levels of α‐1‐antitrypsin, zonulin, calprotectin, and lactoferrin in the feces of PD patients compared to HS. Additionally, levels of calprotectin and lactoferrin were positively correlated with α‐1‐antitrypsin and zonulin levels, aligning with previous studies that link intestinal inflammation to increased permeability [36, 37]. In summary, intestinal permeability is markedly elevated in PD patients, which may lead to adverse effects such as increased translocation of bacteria (e.g., *E. coli* ) [38] and bacterial products (e.g., lipopolysaccharides) [16] from the gut to the blood. This compromised intestinal permeability likely contributes to a pro‐inflammatory environment in the gut and increases oxidative stress on the ENS.

In our untargeted metabolomics analysis of plasma and fecal samples from PD and HS groups, we identified seven metabolites that are elevated in plasma but not in the stool. The elevations of these seven metabolites in plasma are positively correlated with disruption of intestinal permeability and intestinal inflammation or disease severity. Notably, 3‐Methoxytyrosine (3‐MT), previously linked to PD therapeutic drugs, was excluded from further analysis to avoid potential confounding effects from medication. Our results revealed significantly elevated levels of N‐Acetyl‐L‐tyrosine (NALT) in the plasma of PD patients. Previous research on uremia has classified NALT as a uremic toxin when present at high levels in serum or plasma [39, 40]. Uremic toxins are endogenous molecules that, when not properly cleared by the kidneys, can contribute to kidney damage, neurological deficits, and other health issues [41]. Although specific studies on NALT in the plasma of PD patients are lacking, research on N‐terminal acetylated amino acids suggests that such substances might influence alpha‐synuclein aggregation, potentially accelerating PD progression [42, 43]. Moreover, a study of the cerebrospinal fluid (CSF) of PD patients found elevated levels of N‐acetylated amino acids, which were associated with excitotoxicity and oxidative stress in PD pathology [44]. Recent research has also highlighted the role of abnormal histone acetylation and transcriptional regulation changes in PD through western blot analysis [45, 46]. These findings suggest that the elevated plasma levels of NALT may play a significant role in the pathogenesis of PD. Additionally, cellular oxidative stress and reactive oxygen species (ROS) imbalance are key markers of PD, with excessive ROS production linked to neurotoxicity [47, 48]. Recent studies have identified NALT as an endogenous stress response factor that, under stress conditions, can influence ROS release from mitochondria. While low levels of ROS can trigger cytoprotective responses, high levels can be harmful [49]. Thus, we hypothesize that elevated NALT in the plasma of PD patients may lead to excessive ROS release from mitochondria, contributing to neurotoxicity and exacerbating PD progression.

Propyl gallate (PG) is a polyphenol known for its antioxidant properties. Over the past decade, research has highlighted the potential of polyphenols to prevent or mitigate neurodegenerative diseases in both in vivo and in vitro models [50, 51]. However, our study identified a significant increase in PG levels in the plasma of Parkinson's disease (PD) patients. This elevation was positively correlated with disease severity, motor symptoms, and constipation, which seems inconsistent with previous research findings. Notably, there are no prior reports on plasma PG levels in PD patients for comparison. PG is a widely used antioxidant, yet its toxicological effects warrant further investigation [52]. Ham et al. [53] demonstrated that PG could induce male infertility in mice by causing mitochondrial dysfunction, disrupting calcium homeostasis, and inhibiting genes crucial for testicular development. Additionally, PG has been shown to block trophoblast invasion by inducing mitochondrial dysfunction and cell apoptosis [54]. These findings suggest that PG‐induced mitochondrial dysfunction is a key pathway for its toxic effects [52]. Furthermore, PG has been reported to exhibit pro‐oxidant effects, causing DNA strand breaks in the presence of Cu(II) [55]. Depending on dosage and exposure duration, PG can impair the activity of superoxide dismutase and catalase, leading to increased reactive oxygen species (ROS) accumulation [56]. As a common food additive, PG is metabolized by the liver and kidneys. Despite efforts to control dietary differences by selecting spouses who have lived with the patients for extended periods, some variability remains. We hypothesize that the impaired intestinal barrier in PD patients may lead to increased absorption of PG into the bloodstream, where it accumulates and potentially exacerbates PD progression by inducing mitochondrial dysfunction and ROS accumulation. Further animal studies are needed to validate these mechanisms.

We also detected elevated levels of 3,4‐dihydroxyphenylglycol O‐sulfate in the plasma of PD patients, with significant positive correlations to constipation, disease duration, and HAMA scores. Although this metabolite is found in various foods, quantitative reports are lacking. Similar to PG, we suspect that the increase in 3,4‐dihydroxyphenylglycol O‐sulfate is related to intestinal barrier impairment. Capturing these associations is just the initial step of our research, aimed at guiding downstream mechanistic studies. Additionally, we observed significant increases in two other substances—Ethyl N‐(1,4‐thiazinan‐4‐ylcarbothioyl) carbamate and NP‐019988—in the plasma of PD patients, and these substances are not yet annotated in metabolite databases such as HMDB and KEGG. Ethyl N‐(1,4‐thiazinan‐4‐ylcarbothioyl) carbamate was strongly correlated with longer disease duration, more severe MDS‐UPDRS motor scores, and greater non‐motor symptoms (NMSQ). While no direct information was retrieved regarding these specific compounds, our investigation identified that Ethyl N‐(1,4‐thiazinan‐4‐ylcarbothioyl) carbamate belongs to the thiocarbamate class—a group of compounds frequently employed as active ingredients in pesticides known to induce neurotoxicity through mitochondrial dysfunction and ROS generation [57]. In our study, this compound demonstrated significant elevation in the plasma of PD patients and showed a strong correlation with intestinal barrier impairment, leading us to postulate its potential involvement in PD pathogenesis. Similarly, no matching information was found in metabolic databases for NP‐019988. Nonyl phenol (NP), a well‐documented environmental endocrine disruptor widely used in industrial and agricultural applications, has established neurotoxic effects [58]. The precise relationship between NP‐019988 and NP remains unclear. However, if a structural or functional association exists between these compounds, NP‐019988 may potentially enter systemic circulation through dietary exposure. Our study revealed significantly elevated NP‐019988 levels in PD patient plasma, showing notable positive correlations with constipation, disease duration, H‐Y stage, MDS‐UPDRS part II, and PDQ‐39 scores. Based on these clinical correlations and its co‐occurrence with intestinal barrier dysfunction, we hypothesize that NP‐019988 may translocate across the compromised gut barrier into circulation, thereby exerting neurotoxic effects. Although both compounds remain unannotated in current metabolic databases, our study demonstrates their significant elevation in plasma levels and strong association with intestinal barrier dysfunction. Notably, structural analogs of these substances exhibit well‐documented neurotoxic properties. We therefore propose their potential involvement in established PD pathogenic pathways, such as mitochondrial dysfunction, ROS accumulation, and neuroinflammation. As database development progresses and further experiments are conducted, these metabolites will be annotated, potentially providing insights into their mechanisms of action.

The results from our metabolomics analysis provide valuable insights into potential biomarkers for Parkinson's disease (PD) and help uncover biologically plausible disease mechanisms such as excitotoxicity, neuroinflammation, and oxidative stress [59, 60]. With growing recognition of the role of gut microbial‐metabolite pathways in PD, fecal samples have become a common choice for assessing intestinal metabolite levels [12, 61]. However, these levels can be significantly influenced by external factors such as diet, mood, and environmental conditions, which may not fully reflect the disease itself. Interestingly, intestinal metabolites can be absorbed into the bloodstream through the intestinal epithelium and are maintained within physiological ranges through mass action‐driven oxidation [62]. As a result, plasma metabolites are generally more stable and less affected by external variables compared to fecal metabolites. Additionally, plasma sample collection is not restricted by constipation, making it a more reliable option for identifying PD biomarkers and understanding the underlying mechanisms. In fact, dietary habits exert significant influence on both microbial composition and metabolite outcomes, necessitating the establishment of standardized dietary protocols and assessment tools. This study fundamentally addresses dietary variability by selecting healthy spouses who shared long‐term dietary patterns with patients as the control group. Concurrently, we implemented the REAP‐S dietary assessment scale to objectively evaluate the dietary patterns of all participants.

**FIGURE 6:**
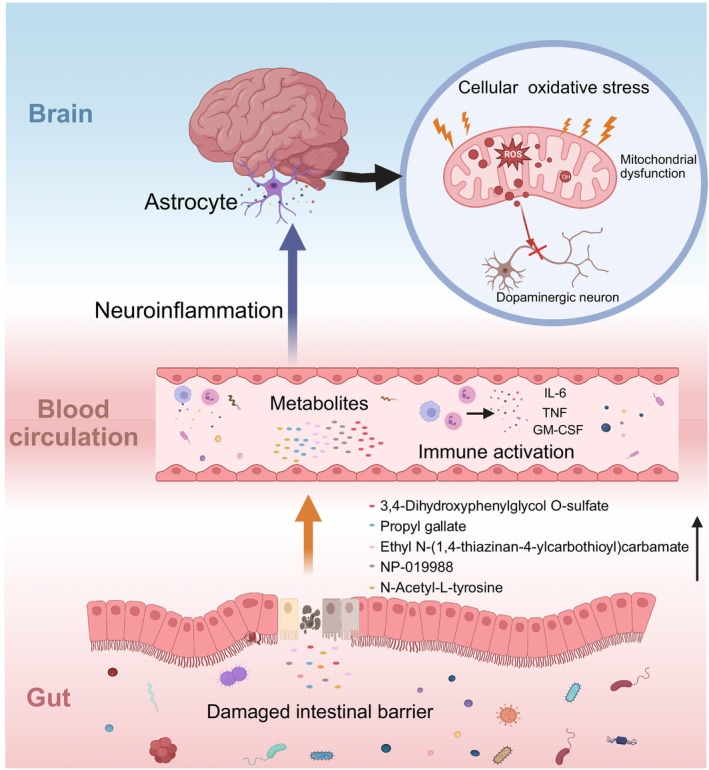
The Potential Mechanism of Action of Differential Metabolites. Five metabolites (NP‐019988, Ethyl N‐(1,4‐thiazinan‐4‐ylcarbothioyl) carbamate, 3,4‐Dihydroxyphenylglycol O‐sulfate, Propyl gallate, and N‐Acetyl‐L‐tyrosine) accumulate in the blood through a compromised intestinal barrier. It is speculated that these substances further induce mitochondrial dysfunction, leading to the death of dopaminergic neurons.

In our study, we identified five metabolites, including 3,4‐dihydroxyphenylglycol O‐sulfate, Ethyl N‐(1,4‐thiazinan‐4‐ylcarbothioyl) carbamate, N‐acetyl‐L‐tyrosine, propyl gallate, and NP‐019988, as potential biomarkers for PD. Notably, propyl gallate and N‐acetyl‐L‐tyrosine, which have been documented in other studies, showed high predictive potential for PD occurrence, with an AUC value reaching 0.91. We speculate that these five substances likely enter the bloodstream through the compromised intestinal barrier in Parkinson's disease (PD) patients and accumulate therein. This accumulation may lead to mitochondrial dysfunction, which in turn triggers an increase in reactive oxygen species (ROS). The elevated levels of ROS can further induce neurotoxicity, ultimately affecting the neurological functions of PD patients and playing a significant role in the progression of the disease. This process may elucidate the potential link between these substances and the pathophysiology of PD, providing new directions for future research and treatment (Figure 6). Previous studies have highlighted short‐chain fatty acids (SCFAs) as key biomarkers in PD, given their role in the microbiota‐gut‐brain axis, gut barrier integrity, and gastrointestinal motility [12, 17, 63]. Recent research noted a discrepancy where SCFAs were low in stool but high in plasma, potentially related to increased intestinal permeability [18, 19]. This suggests that when a substance is present in stool without a significant difference between PD and healthy controls but shows a notable difference in plasma, it could indicate passage through a compromised intestinal barrier into the bloodstream. However, the source and destination of fecal metabolites are relatively complex, and the low levels of metabolites in stool may be due to either their less production or excessive degradation by gut microorganisms, while it is insufficient evidence to suggest that its low concentration is due to the increase of absorption into blood caused by intestinal barrier dysfunction. So, we propose that when a substance exists in the stool of PD and HS without difference, but there is a significant difference in plasma based on the damaged intestinal barrier, this difference provides stronger evidence for its passage through the damaged barrier into the bloodstream.

While our predictive model demonstrated excellent discriminative performance (AUC 0.94) in the discovery cohort, several limitations should be acknowledged: (1) This study did not conduct independent cohort validation. While we implemented rigorous internal validation to ensure model robustness, the absence of an external validation cohort means that we are unable to fully assess the model's applicability across different populations. Future research needs to verify the model's performance through multicenter, large‐scale prospective studies to ensure its stability and reliability in diverse settings. (2) We initially accounted for dietary variations by including spouses as controls, but overlooked the fact that long‐term cohabitation leads to similarities in metabolites due to shared dietary and environmental exposures. Therefore, incorporating healthy individuals from outside the household as an additional control group is essential for subsequent studies. (3) Most patients were already on medication, and we did not include a cohort of newly diagnosed patients who had not yet received treatment to assess the impact of medication effects. Including drug‐naive newly diagnosed patients and stratifying long‐term medicated patients by type of medication would more directly demonstrate the impact of medications on metabolites. (4) As a cross‐sectional study, this study limited our observation of the dynamic changes of these metabolites.

## 5 Conclusion

This study is the first to employ untargeted metabolomics to identify metabolites that exhibited no significant differences in stool samples between PD patients and HS but were significantly elevated in the plasma of PD patients. Our findings identified significant metabolite alterations in PD patients and revealed their associations with intestinal barrier dysfunction and clinical characteristics of the disease. Moving forward, we plan to follow up with these patients to record their dietary habits and establish longitudinal cohorts, allowing for a more comprehensive understanding of the changes in metabolites as the disease progresses.

## Author Contributions

Puqing Wang, Jing Tian, Sufang Liu conceived the project. Qiang Zhao performed the analyses. Xianhong Li, Jie Tang, Juan Wang, Yuting Zhao, Zhengting Yang, Xin Pan, and Rui Xiang collected samples. Sufang Liu and Puqing Wang wrote the manuscript.

## Ethics Statement

This study received approval from the Ethical Committee of Biomedical Basic Research of Xiangyang No. 1 People's Hospital (XYYYE20230027), and written informed consent was obtained from all participants.

## Conflicts of Interest

The authors declare no conflicts of interest.

## Supporting information


Data S1.

## Data Availability

The data that support the findings of this study are available on request from the corresponding author. The data are not publicly available due to privacy or ethical restrictions.
