# Supplementary material for: Unraveling the Relation of Parkinson's Disease and Metabolites: A Combined Analysis of Stool and Plasma Metabolites Based on Untargeted Metabolomics Technology
Source: CNS Neurosci Ther. 2025 May 16;31(5):e70424. doi: 10.1111/cns.70424 (PMC12082280; doi:10.1111/cns.70424)
Supplement: Supplementary file 1 — Data S1. [file CNS-31-e70424-s001.zip › 1.Inclusion and Exclusion Criteria .docx]

**Inclusion and exclusion criteria：**

The patients (PD, n = 55) were recruited by two Parkinson's disease specialists according to the Movement Disorder Society Clinical Diagnostic Criteria for PD. Healthy spouses (HS, n = 55) were lived in the same household with PD patients.This approach was to minimize potential confounding factors such as diet, lifestyle, and living conditions.

Patient exclusion criteria: (1) Parkinsonism-plus syndrome or secondary Parkinsonism syndrome; (2) inflammatory bowel syndrome; (3) psychiatric illness; (4) diabetes, gastrointestinal disease, surgical history or infectious diseases; (5) antibiotics / probiotics used for nearly three months.

Healthy spouses (HS, n = 55) were lived in the same household with PD patients. Exclusion criteria for healthy spouses were as follows: (1) obvious digestive diseases (history of gastrointestinal surgery or severe infection); (2) psychiatric illness and neurodegenerative disease; (3) use of antibiotics / probiotics for nearly three months; and (4) history of going out of Xiangyang city (> 5 days) in the last six months.
