# Supplementary material for: Unraveling the Relation of Parkinson's Disease and Metabolites: A Combined Analysis of Stool and Plasma Metabolites Based on Untargeted Metabolomics Technology
Source: CNS Neurosci Ther. 2025 May 16;31(5):e70424. doi: 10.1111/cns.70424 (PMC12082280; doi:10.1111/cns.70424)
Supplement: Supplementary file 1 — Data S1. [file CNS-31-e70424-s001.zip › 2.REAP-S.pdf]

REAPS (Rapid Eating Assessment for Participants - Shortened Version)

CJSegal-Isaacson, EdD RD, Judy-Wylie-Rosett, EdD RD, Kim Gans, PhD, MPH

**In an average week, how often do you:**	**Usually/ Often**	**Sometimes**	**Rarely/ Never**	**Does not apply to me**
1. Skip breakfast?	O	O	O	
2. Eat 4 or more meals from sit-down or take out restaurants?	O	O	O	
3. Eat less than 2 servings of whole grain products or high fiber starches a day? **Serving** = 1 slice of 100% whole grain bread; 1 cup whole grain cereal like Shredded Wheat, Wheaties, Grape Nuts, high fiber cereals, oatmeal, 3-4 whole grain crackers, ½ cup brown rice or whole wheat pasta, boiled or baked potatoes, yuca, yams or plantain.	O	O	O	
4. Eat less than 2 servings of fruit a day? **Serving** = ½ cup or 1 med. fruit or ¾ cup 100% fruit juice.	O	O	O	
5. Eat less than 2 servings of vegetables a day? **Serving** = ½ cup vegetables, or 1 cup leafy raw vegetables.	O	O	O	
6. Eat or drink less than 2 servings of milk, yogurt, or cheese a day? **Serving** = 1 cup milk or yogurt; 1½ - 2 ounces cheese.	O	O	O	
7. Eat more than 8 ounces (see sizes below) of meat, chicken, turkey or fish per day? ***Note**: 3 ounces of meat or chicken is the size of a deck of cards or ONE of the following: 1 regular hamburger, 1 chicken breast or leg (thigh and drumstick), or 1 pork chop.*	O	O	O	Rarely eat meat, chicken, turkey or fish O
8. Use regular processed meats (like bologna, salami, corned beef, hotdogs, sausage or bacon) instead of low fat processed meats (like roast beef, turkey, lean ham; low-fat cold cuts/hotdogs)?	O	O	O	Rarely eat processed meats O
9. Eat fried foods such as fried chicken, fried fish, French fries, fried plantains, tostones or fried yuca?	O	O	O	
10. Eat regular potato chips, nacho chips, corn chips, crackers, regular popcorn, nuts instead of pretzels, low-fat chips or low-fat crackers, air-popped popcorn?	O	O	O	Rarely eat these snack foods O
11. Add butter, margarine or oil to bread, potatoes, rice or vegetables at the table?	O	O	O	
12. Eat sweets like cake, cookies, pastries, donuts, muffins, chocolate and candies more than 2 times per day.	O	O	O	
13. Drink 16 ounces or more of non-diet soda, fruit drink/punch or Kool-Aid a day? ***Note:** 1 can of soda = 12 ounces*	O	O	O	
	**YES**			**NO**
14. You or a member of your family usually shops and cooks rather than eating sit-down or take-out restaurant food?	O			O
15. Usually feel well enough to shop or cook.	O			O

	**1**	**2**	**3**	**4**	**5**
16. How willing are you to make changes in your eating habits in order to be healthier?	**Very willing**				**Not at all willing**
