# Supplementary material for: Unraveling the Relation of Parkinson's Disease and Metabolites: A Combined Analysis of Stool and Plasma Metabolites Based on Untargeted Metabolomics Technology
Source: CNS Neurosci Ther. 2025 May 16;31(5):e70424. doi: 10.1111/cns.70424 (PMC12082280; doi:10.1111/cns.70424)
Supplement: Supplementary file 1 — Data S1. [file CNS-31-e70424-s001.zip › Experiment Methods.pdf]

Experiment Methods

Sample center start to Number each samples to better manage and preserve the them, and the number was used in the experimental testing process.

2.1 Instrument and Reagents

- Low Speed Cryogenic Universal Centrifuge (Centrifuge 5430, Eppendorf)
- Vortex mixer (QL-901, Kylin-Bell Lab Instruments Co.,Ltd， China)
- Ultra pure water meter (Milli-Q Integral, Millipore Corporation, USA)
- Refrigerated Vacuum Concentrator (Maxi Vacbeta, GENE COMPANY)
- Tissue Grinder (JXFSTPRP, Shang hai Xin Ning, China)
- Methanol (A454-4), Acetonitrile (A998-4) were all LCMS level (Thermo Fisher Sci entific, USA)；Ammonia formate (17843-250G，Honeywell Flu ka, USA), Formic acid (50144-50ml, DIMKA, USA). The water was supplied by a ultra pure water meter.
- Internal standard: d3-Leucine, 13C9-Phenylalanine, d5-Tryptophan, 13C3-Progesterone

2.2 Metabo lite extraction

After thawing the sample slowly at 4 °C, weigh 25 mg and put it into 1.5ml Eppendorf tube, add 800 uL extraction solution (methanol: acetonitrile: water =2:2:1, v:v:v, -20 °C precooling) and 10 uL internal standard, add two small steel balls, put them into the tissue grinder for grinding (50 Hz,5min), and after 4 °C water bath ultrasound for 10min. Let the refrigerator stand at 20 °C for 1H. Centrifuge at 25000 g for 15 min at 4 °C. 600 after centrifugation uL supernatant, put it into the freeze vacuum concentrator to drain, and then add 600 uL complex solution (methanol: H2O=1:9, v:v) is redissolved, vortex vibrated for 1min, water bath ultrasonic at 4 °C for 10min, centrifuged at 25000 g at 4 °C for 15min, and the supernatant is placed in the loading bottle. Take 50 supernatants from each sample uL mixing synthetic QC samples are used to evaluate the repeatability and stability of LC-MS analysis process.

2.3 UPLC-MS Analysis

This experiment used Waters UPLC I-Class Plus (Waters, USA) tandom Q Exactive high resolution mass spectrometer (Thermo Fisher Scientific, USA) for separation and detection of metabolites.

Chromatographic conditions: Chromatographic separation was performed on a Waters ACQUITY UPLC BEH C18 column (1.7 μm, 2.1 mm × 100 mm, Waters, USA), and the column temperature was maintained at 45 °C. The mobile phase consisted of 0.1% formic acid (A) and acetonitrile (B) in the positive mode, and in the negative mode, the mobile phase consisted of 10 mM ammonium formate (A) and acetonitrile (B). The gradient conditions were as follows: 0-1

min, 2% B; 1- 9 min, 2%-98% B; 9-12 min, 98% B; 12-12.1 min, 98% B to 2% B; and 12.1-15min, 2% B. The flow rate was 0.35 mL/min and the injection volume was 5 μL.

Mass spectrometry conditions:Using Q Exactive (Thermo Fisher Scientific, USA) perform primary and secondary mass spectrometry data acquisition. The full scan range was 70–1050 m/z with a resolution of 70000, and the automatic gain control (AGC) target for MS acquisitions was set to 3e6 with a maximum ion injection time of 100 ms. Top 3 precursors were selected for subsequent MSMS fragmentation with a maximum ion injection time of 50 ms and resolution of 17500, the AGC was 1e5. The stepped normalized collision energy was set to 20, 40 and 60 eV. ESI parameters were setting as : Sheath gas flow rate was 40, Aux gas flow rate was 10， positive-ion mode Spray voltage(|KV |) was 3.80, negative-ion mode Spray voltage(|KV |) was 3.20，Capillary temperature was 320°C，Aux gas heater temperature was 350°C.

2.4 Metabo lite ion peak extraction and metabo lite identification

After importing the off-line data of mass spectrometry into compound discoverer 3.3 (Thermo Fisher Scientific, USA) software and analyzing the mass spectrometry data in combination with bmdb (BGI metabolome database), mzcloud database and chemspider online database, a data matrix containing information such as metabo lite peak area and identification results will be obtained. After that, the table will be further analyzed and processed.

Software infor：

Compound Discoverer

Version：v.3.3

Paramenter：Parent ion mass deviation: < 5ppm

Mass deviation of fragment ions: < 10ppm

Retention time deviation: < 0.2min
